# Influence of a GSK3β phosphorylation site within the proximal C-terminus of Neurofilament-H on neurofilament dynamics

**DOI:** 10.1242/bio.028522

**Published:** 2017-09-07

**Authors:** Rishel Brenna Vohnoutka, Edward F. Boumil, Yuguan Liu, Atsuko Uchida, Harish C. Pant, Thomas B. Shea

**Affiliations:** 1 Laboratory for Neuroscience, University of Massachusetts Lowell, Lowell, MA 01854, USA; 2 Department of Biomedical and Nutritional Sciences, University of Massachusetts Lowell, Lowell, MA 01854, USA; 3 Department of Neuroscience, Ohio State University, Columbus, OH 43210, USA; 4 Cytoskeletal Protein Regulation Section, National Institute of Neurological Disorders and Stroke, National Institutes of Health, Bethesda, MD 20892, USA

**Keywords:** Axonal cytoskeleton, Neurofilament, Phosphorylation, Protein conformation, Proteolysis

## Abstract

Phosphorylation of the C-terminal tail of the heavy neurofilament subunit (NF-H) impacts neurofilament (NF) axonal transport and residence within axons by fostering NF-NF associations that compete with transport. We tested the role of phosphorylation of a GSK-3β consensus site (S493) located in the proximal portion of the NF-H tail in NF dynamics by transfection of NB2a/d1 cells with NF-H, where S493 was mutated to aspartic acid (S493D) or to alanine (S493A) to mimic constitutive phosphorylation and non-phosphorylation. S493D underwent increased transport into axonal neurites, while S493A displayed increased perikaryal NF aggregates that were decorated by anti-kinesin. Increased levels of S493A co-precipitated with anti-kinesin indicating that reduced transport of S493A was not due to reduced kinesin association but due to premature NF-NF interactions within perikarya. S493D displayed increased phospho-immunoreactivity within axonal neurites at downstream C-terminal sites attributable to mitogen-activated protein kinase and cyclin-dependent kinase 5. However, S493D was more prone to proteolysis following kinase inhibition, suggesting that S493 phosphorylation is an early event that alters sidearm configuration in a manner that promotes appropriate NF distribution. We propose a novel model for sidearm configuration.

## Abstract (precis)

**Summary:** We demonstrate that phosphorylation of a critical site regulates neurofilament transport, proteolysis and interaction with other axonal cytoskeletal elements, and present evidence that it does so by altering protein conformation. This article has an associated First Person interview with the first author of the paper as part of the supplementary information.

## INTRODUCTION

The cytoskeleton provides structural support to axons allowing for axonal outgrowth and for maintenance of synaptic connections formed by outgrowing axons (Luo, 2002; Barnes and Polleux, 2009; Kapitein and Hoogenraad, 2011). Mammalian neurofilaments (NFs), a major constituent of the axonal cytoskeleton, are composed of three subunits termed NF-light, -medium, and -heavy (NF-L, NF-M, and NF-H, respectively) in reference to their relative molecular mass (Julien and Mushynski, 1998), as well as α-internexin and peripherin (Yuan et al., 2006, 2012).

The C-terminal tail domains of NF-H and NF-M undergo extensive post-translational modification by phosphorylation (Julien and Mushynski, 1982; Carden et al., 1985; Jones and Williams, 1982; Lee et al., 2014). Phosphorylation of the NF-H tail domain induces divalent cation-mediated NF-NF associations. These associations both regulate the formation of the axonal NF cytoskeletal array and foster at least transient withdrawal of NFs from the transporting pool (Leterrier and Eyer, 1987; Gou et al., 1998; Chen et al., 2000; Yabe et al., 1999, 2001; Uchida et al., 2009; Ackerley et al., 2003; Jung and Shea, 1999; Shea and Lee, 2013; Lee et al., 2014). C-terminal phosphorylation also precludes NF proteolysis and increases NF residence time within axons (Lee et al., 2014; Pant and Veeranna, 1995; Greenwood et al., 1993; Nixon, 1993; Goldstein et al., 1987; Fiumelli et al., 2008; Grant et al., 2001). These collective phosphorylation events are mediated by an interactive network of kinases and phosphatases that regulate NF transport and incorporation into the axonal cytoskeleton including: cyclin-dependent kinase (cdk5), mitogen activated kinases (MAPKs), casein kinase 1 and 2 (CK1 and CK2), glycogen synthase kinase 3α and 3β (GSK3α and GSK3β), p38 MAPK, c-Jun N-terminal kinase (JNKs) and protein phosphatases 1, 2A and 2B (PP1, PP2A, PP2B, respectively) (Lee et al., 2014; Pant and Veeranna, 1995, and references therein).

A GSK3β consensus sequence is located at the beginning of the NF-H tail domain; however, the consequences of phosphorylation of this site remain unclear (Sasaki et al., 2002, 2009). To probe the impact of S493 phosphorylation on NF dynamics, we generated phosphomimetic (S493D) and phosphorylation-deficient (S493A) mutations of this site. As demonstrated herein, pseudo-phosphorylation of S493 increased downstream NF-H C-terminal phosphorylation events and increased NF-H accumulation within axonal NFs, while prevention of S493 phosphorylation instead fostered perikaryal NF aggregates. However, pseudo-phosphorylation of S493 also rendered NF-H more susceptible to proteolysis. Our findings suggest that S493 phosphorylation occurs relatively early during the phosphorylation cascade of the NF-H tail domain, and that S493 phosphorylation induces one or more conformational changes in the NF-H C-terminal tail domain that regulate NF dynamics.

## RESULTS

**Fig. 1.**
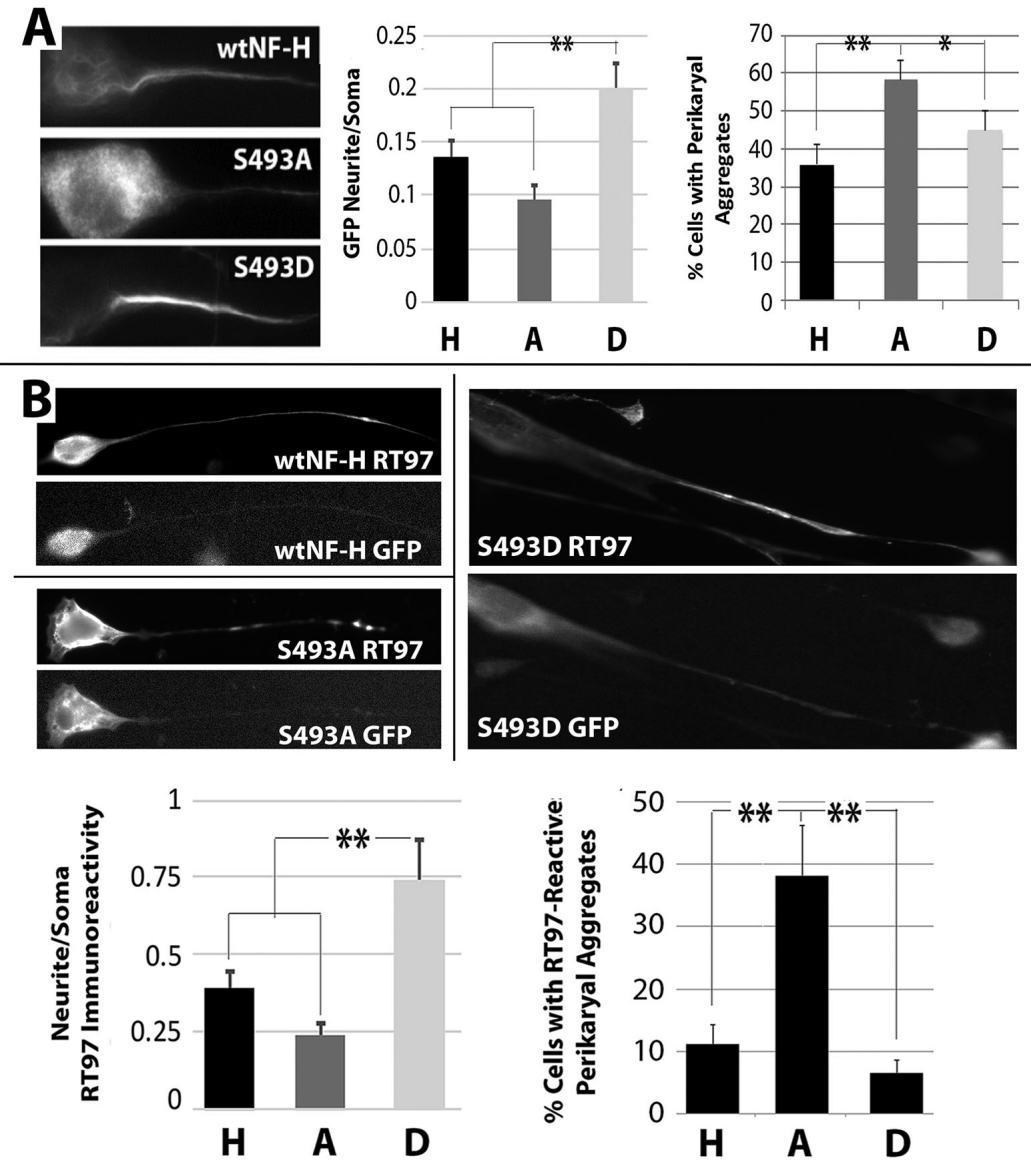
**Differential distribution of GFP-tagged constructs.** (A) Representative images of fluorescent cells expressing wtH, S493A, or S493D. Note S493D-cells display increased axonal fluorescence while S493A-cells display increased perikaryal fluorescence versus wtH-cells. The accompanying graphs present: quantification of the axonal/somal GFP-fluorescence ratio (left) and the mean percentage of cells (right) displaying perikaryal aggregates of GFP-tagged wtH, S493A, or S493D (labeled as H, A, and D, respectively) from three independent experiments. (B) Representative images of cells expressing wtH, S493A, or S493D and probed with anti-phospho-H antibody (RT97) along with corresponding GFP-fluorescent images to confirm transfection. The accompanying graphs present the ratio of the neurite/somal phospho-H immunoreactivity (left) and the mean percentage of cells displaying phospho-H-immunoreactive perikaryal aggregates from ≥3 independent experiments. Note the increased percentage of S493A-cells displaying perikaryal phospho-H aggregates. Error bars in A and B represent ±s.e.; **P<0.05 (ANOVA).

Following knockdown of endogenous NF-H and expression of NF constructs, a significant increase in S493D was observed within axonal neurites versus that of wtH or S493A, while reduced levels of S493A were observed within axonal neurites verus wtH (Fig. 1A). Cells expressing S493A displayed a significant increase in cells exhibiting GFP-tagged perikaryal aggregates versus those of cells expressing wtH or S493D (Fig. 1A). These findings suggest that phosphorylation of S493 promotes transport of NF-H into and along axons, and/or accumulation within axons, while the prevention of S493 phosphorylation disrupts normal transport and results in aberrant accumulation of NF-H within soma.

To examine the potential effect of S493 phosphorylation on downstream NF-H tail domain phosphorylation, immunoblots were probed with monoclonal antibody RT97, which reacts with a conformationally dependent phospho-epitope located within the NF-H tail downstream of S493 (Veeranna et al., 2008). Cytoskeleton-associated RT97 was significantly increased in cells expressing S493D versus S493A and wtH (Fig. 1B), suggesting that phosphorylation of the S493 site potentiates incorporation of NFs into the cytoskeleton. Immunofluorescent analysis displayed more phospho-H in axons of cells expressing S493D versus those expressing wtH or S493A. Conversely, cells expressing S493A displayed an increase in RT97-reactive perikaryal aggregates versus cells expressing wtH or S493D. Moreover, cells expressing S493D displayed a decrease in perikaryal aggregates versus wtH cells (Fig. 1B).

**Fig. 2.**
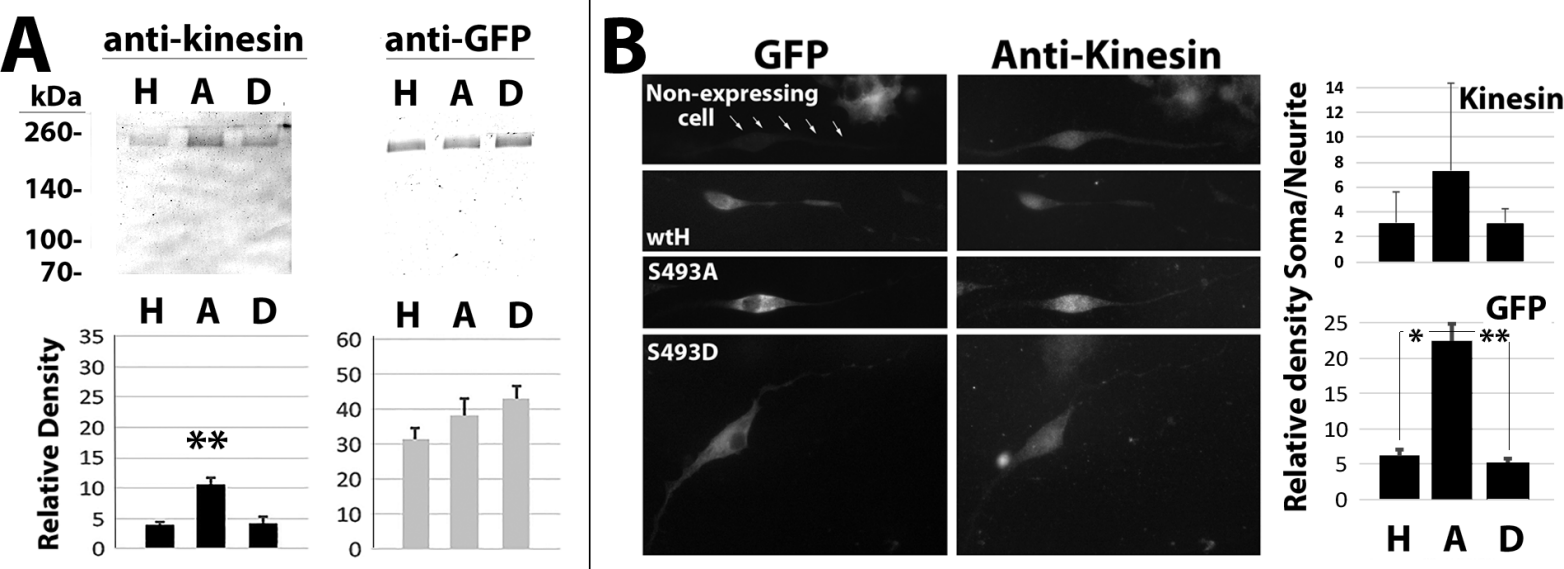
**S493A exhibits an increased association with kinesin.** (A) Nitrocellulose replicas, probed with the indicated antibodies, of NF-H immunoprecipitated from Triton-soluble fractions with anti-GFP or co-immunoprecipitated with anti-kinesin. H, A and D indicate wtH-, S493A- and S493D-cells, respectively. The accompanying graphs present quantification of immunoreactive material corresponding to full-length NF-H within precipitated fractions probed with anti-GFP (mean±s.e. from 3 independent experiments). Note increased co-precipitation of S493A versus wtH and S493D by anti-kinesin. **P<0.05; ANOVA. (B) Representative images of transfected cells following reaction with anti-kinesin along with corresponding images of the distribution of GFP. The first image presents a representative cell from a transfected culture displaying no detectable GFP but with the minor ‘leak’ of fluorescent signal (arrows; compare with the intensity of the cell present in the upper right). The accompanying graph presents quantification of somal/neurite kinesin or GFP immunoreactivity in arbitrary densitometric units. Values represent the mean±s.e. Considerable variation was displayed among cells but note the increase in somal/neurite ratio of both kinesin and GFP for cells expressing S493A. **P<0.05, *P<0.1; ANOVA.

Association of NF-H, S493A, and S493D with kinesin (which transports NF-H into and along axons; Yabe et al., 1999, 2000) was assessed via immunoprecipitation of motor proteins followed by immunoblot analyses to detect any co-precipitated NF-H (Fig. 2A). Unexpectedly, anti-kinesin co-precipitated significantly more S493A than wtH or S493D, suggesting that reduction in axonal S493A was not derived from restriction in the ability of S493A to associate with kinesin. The increased association of S493 with kinesin was unlikely to be due to an undetermined artifact, since similar levels of all three constructs were precipitated by anti-GFP (Fig. 2A).

Immunofluorescent analyses of the distribution of kinesin within soma and neurites were conducted to address further the relative increase in association of S493A with kinesin. Considerable variation was displayed among cells expressing all constructs, but we noted an increase in kinesin within soma versus neurites for cells expressing S493A (Fig. 2B). These findings suggest that the increased co-precipitation of S493A by anti-kinesin may have resulted at least in part by sequestering of kinesin by S493A aggregated within soma, since NF aggregates have previously been shown to bind motor proteins including kinesin (Shea and Beaty, 2007; Toyoshima et al., 1998).

**Fig. 3.**
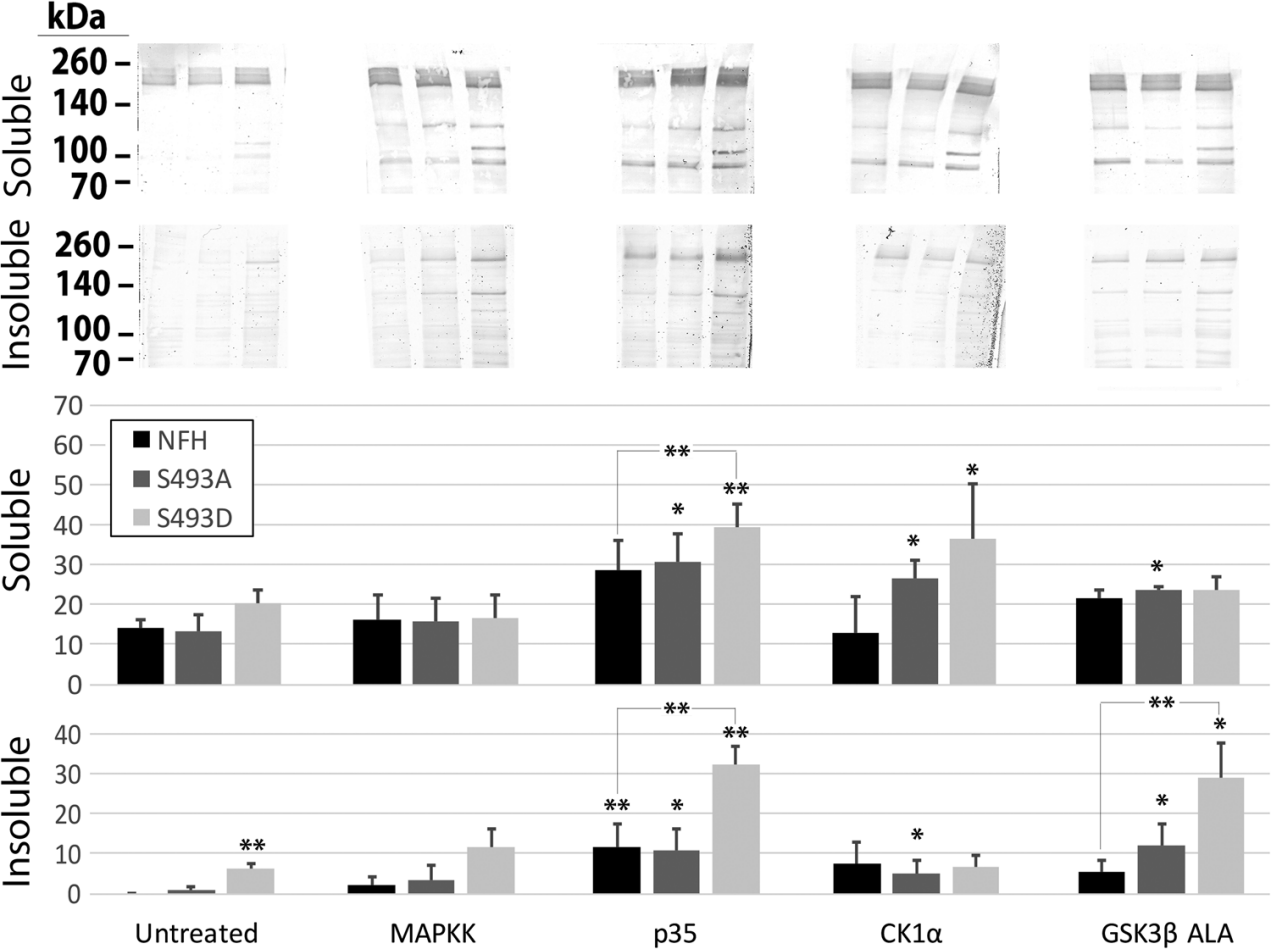
**Upregulation of NF kinases and inhibition of calpain increase NF-H steady-state levels.** Nitrocellulose replicas probed with anti-GFP of Triton-soluble and -insoluble fractions from cells of cells expressing wtH, S493A or S493D (H, A, D, respectively) and co-transfected with the indicated kinases. Note that upregulation of cdk5 (by expression of its activator, p35), and GSK3β increased insoluble S493D, while upregulation of CDK5 and CK1α increased soluble S493D. Breakdown products (not included in quantification) were increased in all samples following calpain inhibition. Error bars indicate mean±s.e. **P<0.05, *P<0.1; ANOVA.

To examine directly whether or not phosphorylation of S493 increased NF-H steady-state levels, and, if so, the responsible mechanism(s), we manipulated the activity of a number of known NF kinases (MAPK, cdk5, CK1α, and GSK3β) in cells expressing wtH, S493A and S493D (Fig. 3A). Upregulation of cdk5 activity (by expression of its activator p35; e.g. Lee et al., 2014) and Ck1α each increased Triton-soluble S493D. Upregulation of CDK5, Ck1α and GSK3β each increased Triton-soluble S493A, but not significantly. Consistent with the increased levels of S493D observed within axonal neurites, increased levels of S493D were observed within the Triton-insoluble fraction versus wtH or S493A prior to kinase manipulation, and were significantly increased following upregulation of cdk5 and GSK3β (Fig. 3). Triton-insoluble wtH was also increased following upregulation of cdk5, while levels of Triton-insoluble S493A were increased (but not significantly) following upregulation of cdk5, Ck1α and GSK3β. The relatively larger effects of kinase upregulation on S493D levels suggests that phosphorylation of S493 increases the ability of the NF-H tail domain to undergo additional phosphorylation events.

**Fig. 4.**
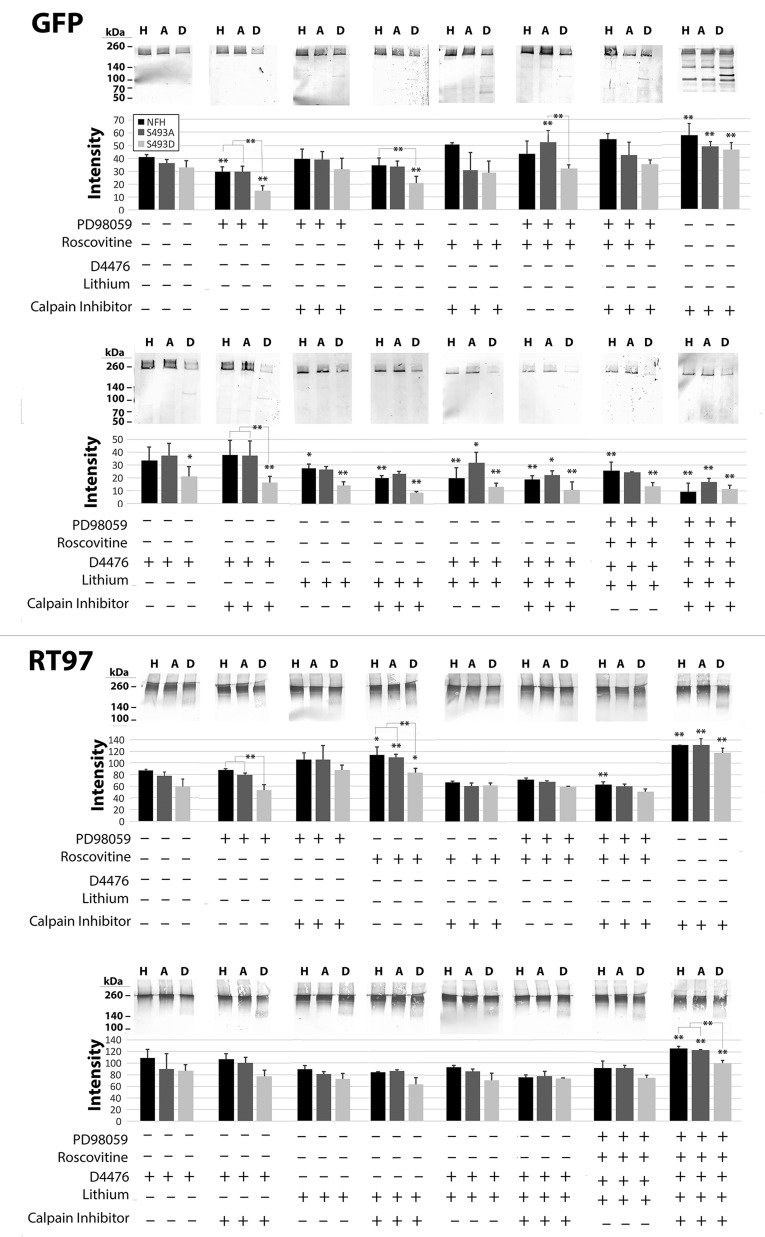
**Phosphorylation of S493 increases the susceptibility of NF-H to proteolysis.** Nitrocellulose replicas probed with anti-GFP or RT97 of Triton-soluble fractions from cells of cells expressing wtH, S493A or S493D (H, A, D, respectively) ±inhibitors active against MAPK (PD98059), CDK5 (Roscovitine), CK1α (D4476), GSK3β (Lithium), and calpain (calpain inhibitor III) as indicated. The accompanying graphs present quantification of GFP, and phospho-H immunoreactivity corresponding to full-length NF-H (mean±s.e. from three independent experiments). Note that GFP-S493D exhibits the largest reduction following inhibition of all NF kinases individually: calpain inhibitor prevented the decline observed following inhibition of MAPK or CDK5. RT97 immunoreactivity was not reduced by kinase inhibition, but was increased by inhibition of cdk5, calpain, and simultaneous inhibition of all four kinases+calpain. Inhibition of calpain increased soluble levels of all three constructs. Breakdown products (not included in quantification) were increased in all samples following calpain inhibition. **P<0.05, *P<0.1; ANOVA.

**Fig. 5.**
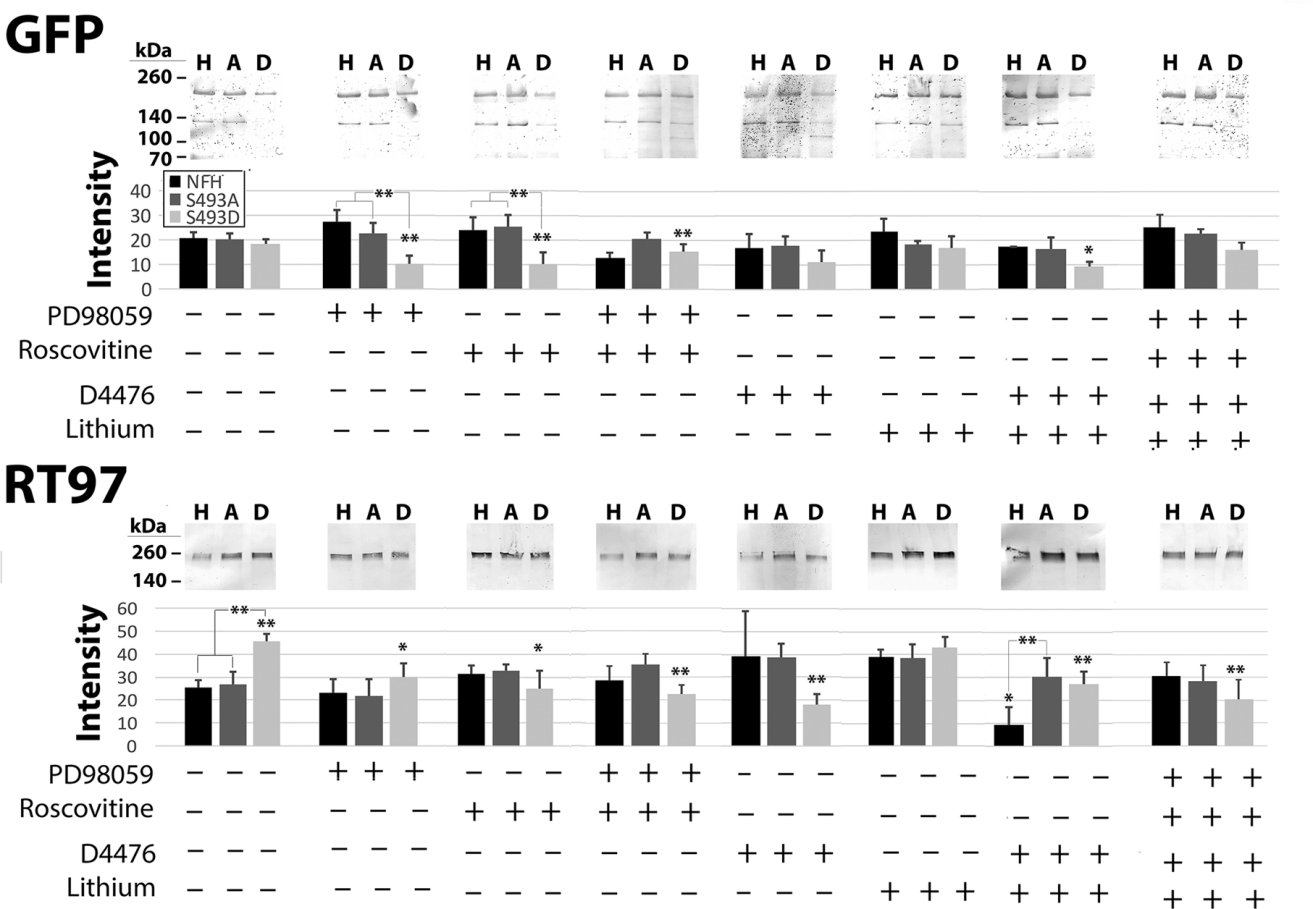
**Phosphorylation of S493 increases the susceptibility of NF-H to proteolysis.** Panels present nitrocellulose replicas probed with anti-GFP or anti-phospho-H (RT97) of Triton-insoluble fractions from cells of cells expressing wtH, S493A or S493D (H, A, D, respectively) ±inhibitors active against MAPK (PD98059), CDK5 (Roscovitine), CK1α (D4476) and GSK3β (Lithium). The accompanying graphs present quantification of wtH, S493A, and S493D, and phospho-H (RT97) (mean±s.e. from three independent experiments). Phospho-H was depleted in S493D-cells to a greater extent than in wtH- or S493A-cells following inhibition of CDK5, CDK5+MAPK, CK1α, GSK3β+CK1α, or all four kinases. By contrast, CK1α inhibition increased phospho-H in wtH- and S493A-cells. Inhibition of GSK3β or GSK3β+CK1α depleted phospho-H in wtH-cells. **P<0.05, *P<0.1; ANOVA.

Since NF steady-state levels are regulated by proteolysis, and proteolysis is suppressed by MAPk and cdk5 phosphorylation events (Lee et al., 2014 and references therein), we examined the consequences of manipulation of calpain activity. Calpain inhibition did not alter levels of Triton-insoluble wtH, S493A or S493D (not shown), but significantly increased Triton-soluble levels of all three constructs, confirming that steady-state levels of all three constructs were regulated by calpain (Figs 4 and 5). GFP-immunoreactive NF-H proteolytic fragments migrating at approximately 130-140 kDa, 110-120 kDa, and 100 kDa were observed (Figs 4 and 5). Retention of GFP-immunoreactivity in all breakdown products indicates proteolytic cleavage from the C-terminal end of the protein, as the GFP-tag is N-terminally located. Upregulation of all NF kinases increased the number of proteolytic fragments following expression of all constructs (Fig. 3). Only the largest of these fragments retained the RT97 epitope (located within the C-terminal tail and generated by MAPk and cdk5) (Veeranna et al., 2008) (Fig. 4). Calpain inhibition further preserved these proteolytic fragments, suggesting that these fragments otherwise underwent continued degradation by calpain-mediated proteolysis (Figs 4 and 5). The 110-120 kDa fragment was only present in cells expressing S493D (Fig. 4), suggesting that phosphorylation of S493D altered sidearm proteolysis and/or placed the sidearm at increased risk for proteolysis; this particular fragment could be generated by cleavage within the MAPK and/or CDK5 consensus sites, since inhibition of these kinases promotes NF-H proteolysis (Pant, 1988; Lee et al., 2014).

We next examined the consequence of inhibition of the above NF kinases by treatment of cells with pharmacological agents active against each kinase (Lee et al., 2014) on total (GFP-immunoreactive) and phosphorylated (RT97-immunoreactive) levels of each construct (Fig. 4). This yielded results more complex than those observed following kinase upregulation, since previously-phosphorylated NF-H may not be affected by the relatively short-term (4 h) inhibition of kinases as utilized herein. Inhibition of MAPK reduced levels of GFP-wtH and GFP-S493D; S493D was reduced to a greater extent than wtH. Inhibition of cdk5 reduced levels of GFP-S493D but not those of GFP-wtH or GFP-S493A. Inhibition of calpain prevented these reductions, suggesting that they were mediated by calpain. Unexpectedly, simultaneous inhibition of both MAPK and cdk5 prevented reductions observed for GFP-wtH and GFP-S493D following inhibition of either kinase alone, and statistically increased levels of GFP-S493A; the potential role of phospho-mediated changes in tail conformation underlying these findings will be discussed below. Inhibition of CK1α reduced levels of GFP-S493D. Inhibition of GSK3β reduced levels of GFP-wtH and GFP-S493D. Combined inhibition of GSK3β+CK1α further reduced levels of GFP-wtH. Consistent with prior studies (Lee et al., 2014), inhibition of calpain did not prevent decreases in NF-H following inhibition of GSK3β±CK1α. Simultaneous inhibition of all four kinases reduced GFP-wtH and GFP-S493D, and this reduction was not prevented by inhibition of calpain. Since reduction of constructs following inhibition of MAPK or cdk5 was prevented by inhibition of calpain, these findings suggest that proteolysis induced by separate or simultaneous inhibition of GSK3β±CK1α may be derived from one or more proteases other than calpain.

Levels of RT97-immunoreactivity were also examined (Fig. 4). Inhibition of MAPk decreased levels of RT97-immunoreactive S493D. Unexpectedly, inhibition of cdk5 increased levels of RT97-immunoreactive wtH, S493A and S493D. This increase was attenuated by simultaneous inhibition of MAPk and by inhibition of calpain. In this regard, the kinases involved in NF-H phosphorylation form a complex hierarchy in which inhibition or increase in one NF kinase affects the activity of other kinases and phosphatases (Lee et al., 2014). Levels of all three constructs were unchanged following inhibition of GSK3β±Ck1α or following simultaneous inhibition of all four constructs. Consistent with increased levels of all three constructs following inhibition of calpain as monitored by GFP, levels of RT97-immunoreactive wtH, S493A and S493D were increased following inhibition of calpain. However, unlike the decreases in GFP levels observed following simultaneous inhibition of calpain and all four kinases, the increases in RT97-immunoreactive wtH, S493A and S493D following inhibition of calpain persisted despite inhibition of all four NF kinases. This is likely due to selective preservation of subunits that had already been phosphorylated and retained their respective phosphates during our short-term inhibition regimen.

We next examined the impact of kinase inhibition on subunits already incorporated into the Triton-insoluble cytoskeleton (Fig. 5). GFP-S493D levels were significantly reduced following inhibition of MAPk and/or cdk5 and slightly reduced following inhibition of CK1α. RT97-immunoreactive S493D was reduced following inhibition of all four kinases individually and in combination. Combined inhibition of GSK3β+CK1α decreased RT97-immunoreactive wtH. Consistent with prior studies on NF phosphorylation (Lee et al., 2014), inhibition of calpain did not reduce RT97-immunoreactive levels of any NF-H construct within the Triton-insoluble fraction (not shown).

**Fig. 6.**
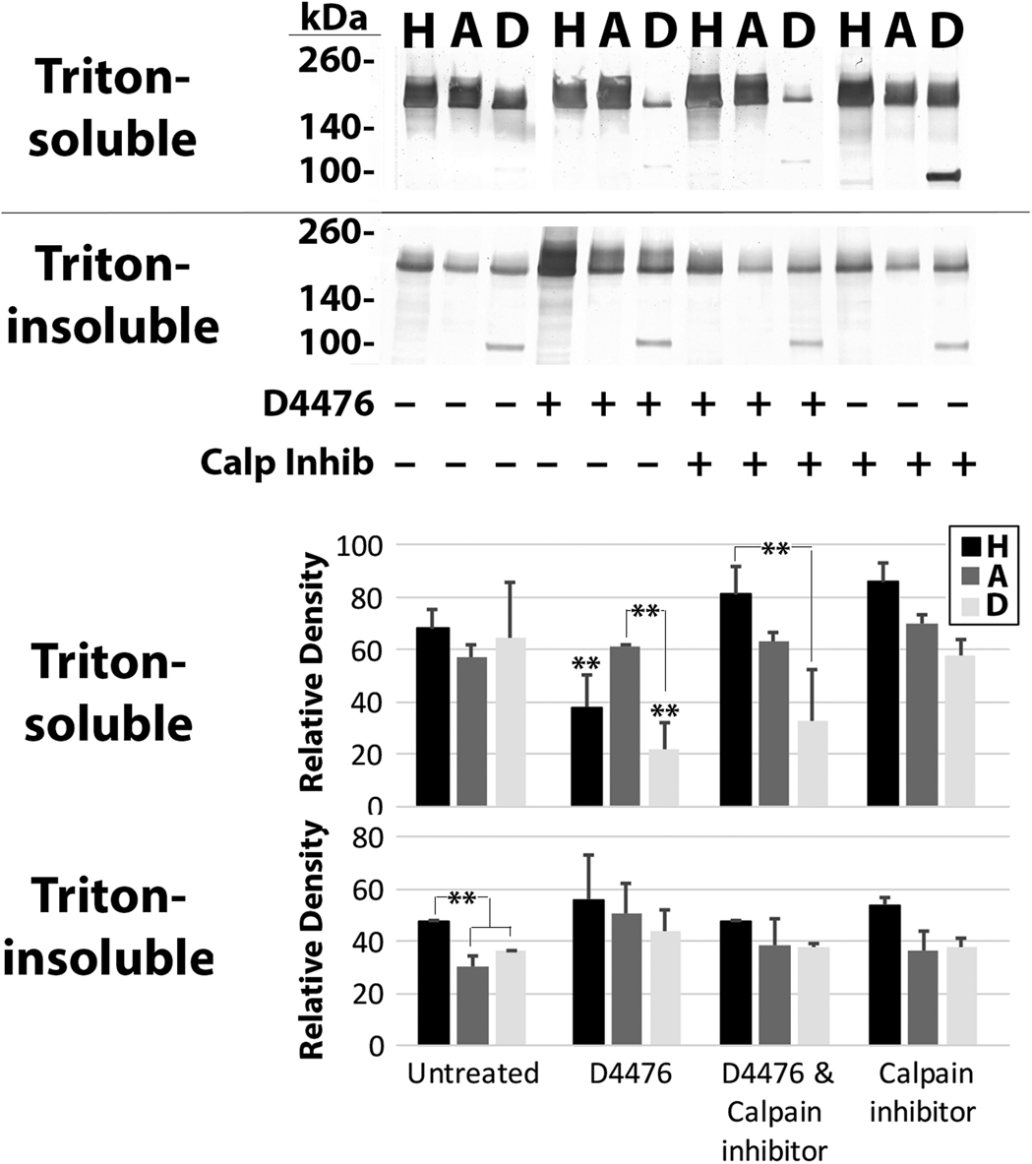
**Phosphorylation of the proximal tail region by CK1α susceptibility of S493D to proteolysis.** Panels present nitrocellulose replicas probed with anti-GFP of Triton-soluble and -insoluble fractions from cells expressing wt-HΔ187, S493AΔ187-cells, and S493DΔ187 (H, A, D, respectively). The accompanying graphs present quantification of GFP immunoreactivity associated with full-length Δ187 (mean±s.e. from three experiments). Levels of soluble wtHΔ187 and S493DΔ187 were reduced by addition of D4476; addition of calpain inhibitor prevented depletion of wtHΔ187 but not S493DΔ187. Levels of insoluble S493DΔ187 was reduced following addition of D4476+calpain inhibitor. **P<0.05, *P<0.1; ANOVA.

Inhibition of CK1α selectively depleted Triton-soluble and -insoluble levels of S493D, but not those of wtH or S493A (Figs 4 and 5). Four CK1α consensus sites are located in the proximal portion of the tail, while many more are located within the terminal 187 amino acid residues (Chen et al., 2000). In initial efforts to localize the impact of inhibition of CK1α on depletion of S493D, we expressed constructs of wt-H, S493A and S493D in which the terminal 187 amino acids were deleted (wtHΔ187, S493AΔ187, and S493DΔ187), which therefore retained only the proximal CK1α consensus sites. Inhibition of CK1α reduced Triton-soluble levels of wtHΔ187 and S493DΔ187 (Fig. 6); while simultaneous inhibition of CK1α+calpain prevented the reduction in levels of wtHΔ187, but did not prevent the reduction in S493DΔ187. These findings suggest that the increased susceptibility of S493D to proteolysis following CK1α inhibition is derived from inhibition of phosphorylation of one or more of the proximal CK1α consensus sites. Inhibition of CK1α did not statistically alter Triton-insoluble levels of any construct, consistent with the likelihood that NF-H incorporated into the Triton-insoluble cytoskeleton had already undergone phosphorylation by this and other NF kinases (Lee et al., 2014).

## DISCUSSION

Our findings suggest that phosphorylation of S493 induced one or more C-terminal conformational changes since (1) S493D underwent significantly larger increases than did S493A or wt-NF-H following co-expression of NF kinases, and (2) S493D underwent significantly more calpain-mediated proteolysis than S493A or wt-H. Breakdown products for all three constructs were observed at 130-140 kDa and at 100 kDa. S493D displayed an additional fragment migrating at 110-120 kDa. However, only the largest (130-140 kDa) species retained the RT97 epitope. GFP-tagged NF-H in which the terminal 187 amino acids have been deleted migrates at approximately 150 kDa and is reactive with RT97, while the GFP-tagged NF-H rod lacking the entire sidearm migrates at 115-120 kDa and does not react with RT97 (Lee et al., 2014; Lee and Shea, 2014). Retention of N-terminally located GFP in all S493D proteolytic fragments, coupled with loss of the C-terminal RT97 epitope in all but the largest (130-140 kDa) fragment, confirmed proteolytic cleavage of C-terminal but not N-terminal regions. Moreover, migration on SDS-gels demonstrates that some of the S493D proteolytic fragments were larger than (130-140 kDa) or as large as (110-120 kDa) the NF-H rod, confirming that multiple cleavage events occurred within the C-terminal tail. The S493D-specific 110-120 kDA fragment was not observed following calpain inhibition. Phosphorylation of S493 therefore likely induces one or more conformational changes that increase accessibility of the NF-H C-terminal tail to NF proteases as well as NF kinases. In this regard, the region surrounding S493 is a so-called ‘PEST’ motif (a region rich in proline, glutamate, serine and threonine residues). PEST motifs are associated with proteasome and calpain-mediated degradation (Shumway et al., 1999). This may underlie why phosphorylation of S493 and/or the regional CK1α consensus sites may be critical in regulation of NF-H levels.

Phosphorylation of S493 likely represents an early event in overall NF tail phosphorylation, since (1) S493 is phosphorylated prior to the appearance of immunoreactivity towards monoclonal antibody SMI-31 (which recognizes an early C-terminal phospho-epitope; Sasaki et al., 2002, 2009; Sánchez et al., 2000), and (2) expression of S493D increased NF-H accumulation of phospho-NFs within axonal NFs, corresponding to the normal distribution of extensively phosphorylated NFs. Conversely, expression of S493A fostered perikaryal phospho-NF aggregates, reminiscent of the perikaryal phospho-NF accumulations within perikarya following increased cdk5-mediated NF-H phosphorylation or inhibition of phosphatase activity without upregulation of S493 phosphorylation (Shea et al., 2004; Lee et al., 2014; Sasaki et al., 2006; Kushkuley et al., 2010; Holmgren et al., 2012). Perikaryal aggregates sequester motor proteins including kinesin (Shea and Beaty, 2007; Toyoshima et al., 1998), which likely underlies the increased levels of kinesin within perikarya of cells expressing S493A as well as increased recovery of S493A by anti-kinesin co-precipitation. The full influence of prevention of phosphorylation of S493 is unclear, since S493A did display downstream phosphorylation as evidenced by the presence of RT97, albeit at reduced levels compared to S493D. Failure to phosphorylate S493 prior to downstream phosphorylation may result in an inappropriate conformation of the tail that favors precocious NF-NF associations. This represents an instance where mutagenesis to prevent phosphorylation at a particular site does not provide the physiological opposite to pseudo-phosphorylation. These results also indicate that increased levels of S493D within the axonal cytoskeleton were not due to an increased propensity to associate with kinesin, but rather due to an increased ability to form NF-NF associations within axonal neurites (Lee et al., 2014; Pant, 1988; Goldstein et al., 1987; Greenwood et al., 1993).

**Fig. 7.**
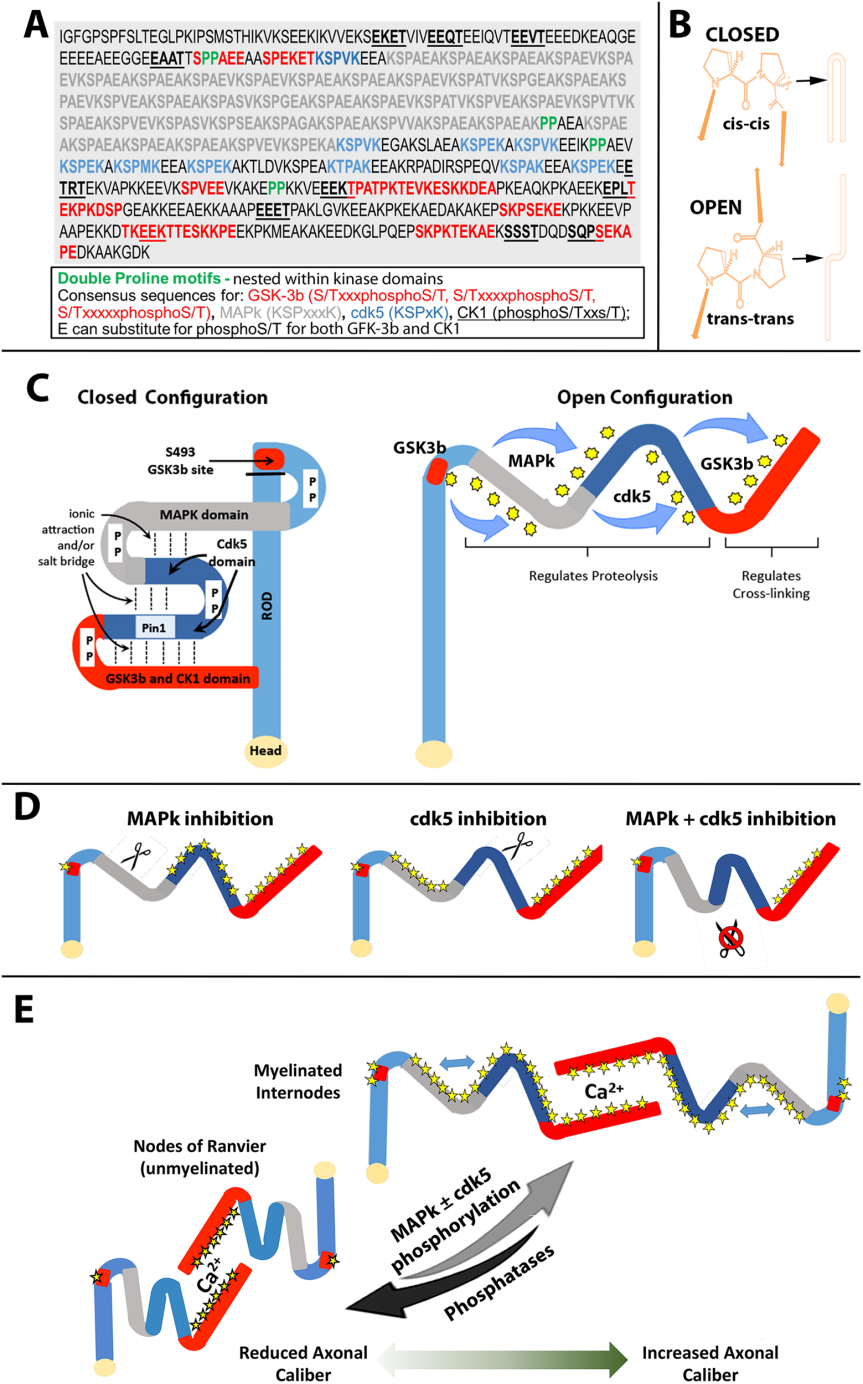
**Proposed model for role of S493 and subsequent phosphorylation events in NF tail configuration.** (A) Amino acid sequence of the rat NF-H C-terminal tail. Consensus sequences for GSK3b are red, those for MAPk are gray, those for cdk5 are blue, and those for CK1a are underscored; note that some GSK3b and CK1a consensus sequences overlap. Found double proline (PP) motifs (indicated in green) are nested within domains consisting of multiple consensus sequences for each of these kinase: the first PP is nested within in the proximal GSK-3b/CK1a domain, the second is within the MAPk domain, the third is within the cdk5 domain, and fourth is within the distal GSK-3b/CK1a domain. S493 is the serine immediately before the first PP motif. (B) Schematic of ‘closed’ and ‘open’ configuration of a peptide resulting from *cis-cis* and *trans-trans* configurations of a double proline motif. (C) Hypothetical closed and open configurations of NF-H. The regions containing consensus sequences for GSK3b/CK1a, MAPk, cdk5 and the proximal GSK3b/CK1a domain S493 (since it is part of a GSK3b consensus sequence) are indicated in red, gray, blue and red, respectively, in all images. With all double-prolines (PP) in *cis-cis* configuration, the non-phosphorylated tail could fold or curve back upon itself. Ionic attractions and/or salt bridges could form between opposing regions of the tail. Repulsive forces resulting from regional phosphorylation (indicated by yellow stars) could convert double-prolines to *trans-trans* configurations and foster tail extension into an open configuration. (D) Phosphorylation of S493 is hypothesized to convert the adjacent double-proline to a *trans-trans* configuration and initiate phospho-dependent tail extension. Extension would increase susceptibility to calpain-mediated proteolysis (indicated by scissors) under conditions where only MAPK or cdk5 were active. Conversely, if neither MAPK of cdk5 were active, their nested double-prolines would remain in *cis-cis* configuration and proteolysis would not occur. (E) Phospho-dependent conversion of the tail to an open configuration and formation of phospho-dependent NF-NF associations. Double arrows depict the hypothesized reversible opening and closing of portions of the tail, resulting from phosphorylation or dephosphorylation of MAPk and cdk5 consensus sequences, coupled with conversion of their nested double-proline motifs between *trans-trans* and *cis-cis* configurations. This regional closure may allow for continued NF-NF association despite closer apposition within the reduced caliber in the nodes of Ranvier.

Proline-peptidyl isomerase 1 (PIN1) alters the configuration of the NF-H tail to allow extensive phosphorylation. Proline residues can exist as *cis* or *trans* isomers, with *cis* isomerization fostering a regional fold or curve in the peptide and *trans* isomerization fostering a more extended conformation (Weiwad et al., 2004). PIN1 disrupts the bond that can form between phosphorylated serine or threonine residues and an immediately adjacent proline in a *cis* form, and fosters a switch of that proline to the more stable *trans* isomer (Lu and Zhou, 2007). Notably, proline-directed kinases, including MAPk and cdk5, cannot phosphorylate serines or threonines that are *cis*-bonded to adjacent proline residues (Weiwad et al., 2004). However, as NF-H C-terminal phosphorylation proceeds, *trans* isomerization by PIN-1 fosters progressive sidearm extension and renders additional phosphorylation sites accessible to kinases (Kesavapany et al., 2007; Rudrabhatla et al., 2008, 2009). In efforts to understand how phosphorylation of S493 might participate in conformational changes, we scrutinized the amino acid sequence of the rat NF-H C-terminal tail (since our construct contained the rat sequence). We noted that S493 was immediately followed by a proline residue (Fig. 7A). While it is clear that PIN1 can expose MAPk and cdk5 sites for phosphorylation, initial phosphorylation of a serine adjacent to a proline must occur to generate the phosphoserine-proline bond recognized by PIN1 (Kesavapany et al., 2007; Rudrabhatla et al., 2008, 2009; Weiwad et al., 2004). Phosphorylation of S493, which is part of a GSK3B consensus sequence rather than a proline-directed consensus site, could represent this initiating phosphorylation event. Notably, S501, which is also immediately followed by a proline residue, is part of an additional GSK3b consensus site in the proximal portion of the NF-H tail (Fig. 7A). Phosphorylation of S501, perhaps along with that of S493, may also serve as an initiating event for the action of PIN1 on the NF-H tail and promotion of downstream MAPk/cdk5 phosphorylation events.

S493 is immediately followed by not one but two proline residues (Fig. 7A). So-called ‘double-proline motifs’ provide a hinge-like structure that dictates regional conformation, encompassing fully ‘closed/folded’ *cis-cis* conformations, fully ‘open/extended’ *trans-trans* conformations, and ‘medial’ *cis-trans* and *trans-cis* conformations. All of these, like the conformation of individual prolines, are strongly influenced by the adjacent amino acid residues (Fig. 7B) (MacArthur and Thornton, 1991). We also noted three additional double-proline motifs within the rat tail domain: the first of which was located within the region containing the consensus sequences for MAPk, the second of which was located within the region containing the consensus sequences for cdk5, and the last of which was located within the region containing the consensus sequences for CK1 and GSK3b (Fig. 7C). Prior to C-terminal phosphorylation, these double-proline motifs could rotate to assume a *cis-cis* conformation, perhaps strengthened by attractive forces among positively and negatively charged regions in overlapping segments of the tail, resulting in a series of ‘accordion-like’ folds or curvatures around the double-proline motifs. C-terminal extension could then be mediated by rotation of the respective double-proline to a *trans-trans* configuration following regional phosphorylation by each respective kinase (Fig. 7C).

Notably, this model provides a possible explanation for the unanticipated observation that, while inhibition of either MAPk or cdk5 resulted in proteolysis of the NF-H C-terminal region of S493D, simultaneous inhibition of both MAPk and cdk5 does not result in calpain-mediated proteolysis. Phosphorylation by MAPk would foster a *trans-trans* configuration of the double-proline motif nested within its consensus sequences, and this would expose a portion of the domain rich in cdk5 sequences (Fig. 7D). Prevention of cdk5-mediated phosphorylation under these conditions would therefore result in calpain-mediated proteolysis, as observed. A similar situation would result following phosphorylation by cdk5 coupled with prevention of phosphorylation by MAPk. The double-proline motif nested within the cdk5 sequences would assume a *trans-trans* configuration, exposing the domain rich in MAPk sequences to calpain-mediated proteolysis. Conversely, inhibition of both MAPk and cdk5 would allow their respective nested double-proline motifs to remain in *cis-cis* configurations, which could potentially shield this region from proteolysis, as observed herein. This ‘closed’ conformation could underlie how NFs lacking KSP phosphorylation can undergo transport into and along axons without proteolytic degradation.

This line of reasoning also accommodates the absence of KSP phosphorylation within the nodes of Ranvier (Hsieh et al., 1994) without proteolytic depletion of NFs. In the absence of MAPk and cdk5-mediated KSP phosphorylation, the second and third double-proline motifs could assume (or retain) *cis-cis* conformations. This is not to suggest that phosphorylation of NFs regulate axonal caliber, but rather that such an ‘intermediary’ sidearm conformation could allow the closer NF-NF spacing observed within the nodes of Ranvier, while protecting NFs from proteolysis. Moreover, any phospho-mediated NF-NF interactions within the nodes could be maintained despite closer apposition of NFs via continued *trans-trans* configuration of the first and fourth double-proline motifs, which are flanked by GSK3β and CK1α consensus sites (Fig. 7E). Notably, Sasaki and colleagues reported persistence of S493 phosphorylation within the nodes of Ranvier despite lack of KSP phosphorylation (Sasaki et al., 2009).

In support of the model suggested herein, a GSK3β consensus sequence analogous to S493 is present in the proximal region of the murine and human NF-H C-terminus (Fig. S1) (Chin and Liem, 1990; Julien et al., 1986; Lees et al., 1988; Robinson et al., 1986). The first double-proline in the murine and human NF-H is also located at the start of the sidearm, prior to the first KSP sequence, while the additional double-proline motifs are nested within tail regions rich in KSP sequences and the terminal region rich in GSK3β/Ck1α consensus sequences (Fig. S1). We did not scrutinize the entire tail sequence of all mammals, but of the 92 mammals for which the NF-H sequence is available, 74% displayed a double-proline motif between the proximal E segment (containing GSK3b and/or CK1a consensus sequences) and before the initial KSP-consensus sequence, and all but three of these were immediately preceded by an S or T as part of a CK1a consensus sequence (Table S1). The model proposed herein is also consistent with the proposed behavior of the NF-H tail domain prior to and following phosphorylation as determined by atomic force microscopy and computer analyses (Brown and Hoh, 1997; Malka-Gibor et al., 2017; Zhulina and Leermakers, 2007, 2009), with the exception that our model encompasses the possibility of a more ordered conformation of the tail prior to phosphorylation.

## MATERIALS AND METHODS

### Cell culture and transfection

Mouse neuroblastoma cells (NB2a/d1) were utilized due to their ease of culture and ability to replicate rapidly while still modeling the dynamics of an axon. NB2a/d1 cells express and phosphorylate all three NF subunits (Shea et al., 1988), and exhibit establishment of Triton-insoluble NFs resembling that observed in axons *in situ* (Shaw and Hou, 1990).

Culture reagents were purchased from Sigma-Aldrich (Natick, MA) unless otherwise indicated. Cells were propagated on either plastic tissue culture dishes or glass coverslips coated with laminin and poly-D-lysine and were maintained in high-glucose DMEM containing 10% fetal bovine serum (Atlanta Biologicals: Flowery Branch, GA, USA) with antibiotics. Axonal neurite outgrowth was induced by addition of 1 mM dibutyryl cyclic AMP (dbcAMP) for 3 days (Shea et al., 1988; Yabe et al., 1999). Cells were transiently transfected 24 h after plating using Polyjet (PJ) transfection reagent (SignaGen: Rockville, MD, USA) according to the manufacturer's instructions with 4 µg total of plasmids (listed below). Cells were incubated with the transfection complex for 4 h after which culture medium was replaced with fresh medium lacking the transfection complex but containing dbcAMP.

Cells were transfected with one of three forms of a construct containing the DNA sequence for *Rattus norvegicus* NF-H tagged on the N-terminus with GFP (Lee et al., 2014): a plasmid containing wild-type NF-H (wtH), a plasmid expressing this sequence in which serine 493 was replaced with alanine (S493A) in order to mimic constitutive non-phosphorylation, and a plasmid in which S493 was replaced with aspartic acid (S493D) in order to mimic constitutive phosphorylation. S493A and S493D constructs were prepared from the GFP-tagged NF-H construct using the QuickChange Lightning Site-Directed Mutagenesis Kit (Agilent Technologies: Santa Clara, CA, USA) according to the manufacturer's instructions. PCR amplification was used to introduce the S493A and S493D mutations using the following primers: 5′GAAGCAGCAACTACGGCTCCCCCTG-CAGAAG-3′ (forward) and 5′-CTTCTGCAGGGGGAGCCGTAGTTGCTGCTT C-3′ (reverse) for generation of the S493A mutation and 5′-GAGAAGAAGCAGCAACTACGTGACCCCCTGCAGA-3′ (forward) and 5′-TCTGCAGGGGGTCACGTAGTTGCTGCTTCT TCT C-3′ (reverse) for generation of the S493D mutation.

Additional cells were transfected with truncated versions of each full-length NF-H construct described above, in which the last 187 amino acids of the protein were removed by insertion of an artificial stop codon as described (Lee et al., 2014). This removed the majority of the GSK3β and CK1α consensus sequences (which are located within the terminal 187 amino acids), and retained only two GSK3β consensus sequences (including the GSK3β consensus site at S493 and at S501) and four proximally located CK1α consensus sites. These constructs are termed wtHΔ187, S493AΔ187 and S493DΔ187, respectively.

For cells intended for use in immunocytochemical and immunoprecipitation experiments (described below), each construct was co-transfected with a plasmid that expressed a shRNA targeting an untranslated region (UTR) at the 5′ end of the endogenous *Mus musculus* NF-H RNA transcript. Only NF-H RNA transcripts expressed by the Nb2a/d1 cells contain UTR sequences, allowing for selective transient knockdown of endogenous NF-H (Lee and Shea, 2014). Prior studies demonstrated that exogenous GFP-tagged NF-H was expressed at levels equivalent to that of endogenous NF-H (Lee and Shea, 2014; co-transfection with this shRNA therefore allowed replacement of endogenous NF-H with appropriate levels of exogenous WT NF-H, S493A, or S493D). shRNA co-transfections were performed with more than three times shRNA construct than that of NF-H construct (3 µg shRNA construct/1 µg NF-H construct) to increase the likelihood that cells displaying GFP had also internalized shRNA.

### Manipulation of NF kinases

Kinase inhibition and overexpression were utilized to elucidate the effects of S493 phosphorylation on downstream NF-H C-terminal tail domain phosphorylation. wtH-cells, S493A-cells, and S493D-cells were treated with the following pharmacological kinase inhibitors singularly or in combination for 4 h prior to harvest on day 3 of differentiation: 10 µM PD98059, 20 µM Roscovitine, 100 µM D4476, and/or 10 mM Li^+^ which are active against mitogen-activated protein kinase kinase 1 (MKK1), cdk5, CK1, and GSK3β respectively (Dudley et al., 1995; Meijer et al., 1997; Cheng and Louis, 1999; Rena et al., 2004). These pharmacological agents will be referred to as inhibitors of their target kinases as listed above for simplicity of writing only, with the recognition that they may exert off-target effects, although prior studies from this laboratory have confirmed their relative specificity (Lee et al., 2014; Chan et al., 2004; Shea et al., 2004).

Additional cultures were subjected to co-transfection with the above NF-H constructs and constructs expressing constitutively-active MAPKK (functional as an upstream MAPK pathway activator) (Li et al., 1999), murine p35 (activator of cdk5; Patrick et al., 1999; generous gift of Harish Pant, NIH, Bethesda, Maryland, USA) murine CK1α (Origene: Rockville, MD, USA), or constitutively-active murine GSK3β (‘GSK3β ALA’; generous gift from Chris Miller, Institute of Psychiatry, King's College, UK; Lee et al., 2014). Co-transfected cultures were incubated with 2 µg of each plasmid (to maintain a total of 4 µg and avoid toxicity). The kinases and activators used within these cells, the plasmids used for expression, and activity following expression using independent activity assays have been well-documented and were therefore not included herein (see Lee et al., 2014 and references therein). Additional cultures also received 10 µM calpain inhibitor III (active against calpain I and II; Jourdi et al., 2009). Calpain inhibitor was added to the culture medium on day 3 of differentiation and any kinase inhibitor was added following 1 h incubation with calpain inhibitor alone. Cells were incubated for an additional 4 h before harvesting.

### Cell harvesting

Cells were harvested after 3 days of differentiation for SDS-PAGE analysis using established methods with minor modifications (Yabe et al., 2001). Cultures were scraped on ice in 50 mM Tris-HCl (pH 6.8) containing 1% Triton X-100, 5 mM EDTA, 1 mM PMSF, a protease inhibitor cocktail (Roche Diagnostics, Indianapolis, IN, USA), and PhosStop (Roche Diagnostics). After scraping, 250 units Dnase1 (Sigma-Aldrich) diluted 1:2 in DNase Activating Buffer [10 mM Tris (pH 7.5), containing 0.25 mM MgCl_2_ and 0.5 mM CaCl_2_] was added, lysates were incubated (30 min on ice), homogenized on ice with a loose-fitting Teflon pestle in a borosilicate glass homogenizer (Thomas Scientific: Swedesboro, NJ, USA) and centrifuged (15,000 ×***g*** for 15 min at 4°C). The resultant (Triton-soluble) supernatants were decanted and (Triton-insoluble) sedimented material was resuspended in the same buffer containing 8 M Urea. Samples were normalized according to protein concentration as determined by Pierce BCA Assay (Thermo Fisher Scientific, Waltham, MA, USA) and diluted in 187.5 mM Tris-HCl (pH 6.8) containing 6% (w/v) SDS, 30% glycerol, 0.03% (w/v) bromophenol blue, and 2% β-mercaptoethanol for SDS-PAGE.

### Immunoprecipitation, gel electrophoresis and immunoblot analysis

Triton-soluble fractions were made 1% SDS and diluted 1:5 (vol/vol) in 60 mM Tris-HCl (pH 7.6) containing 190 mM NaCl, 6 mM EDTA, 1.25% Triton X-100, 1 mM PMSF, protease inhibitor cocktail and PhosStop (Shea et al., 1990). Fractions were normalized with respect to protein concentration by Pierce BCA Assay (Thermo Fisher Scientific) and aliquoted into micro-centrifuge tubes, such that each tube contained an equal volume of lysate and 400 µg of total protein. Protein G-conjugated magnetic beads (25 µl; New England Biolabs: Ipswich, MA, USA) were added to each lysate followed by incubation with orbital agitation for 1 h at 4°C. A magnet was used to precipitate the beads (defined as the pre-precipitate) and the supernatants were transferred to a new tube. Each supernatant received 1:100 dilutions of one of the following antibodies: a rabbit polyclonal antibody directed against ubiquitous kinesin heavy chain (UKHC) antibody (Santa Cruz Biotechnology, Dallas, TX, USA), an antibody directed against dynein (clone 70.1; Sigma-Aldrich), a rabbit polyclonal antibody directed against GFP (Thermo Fisher Scientific). Following incubation with orbital agitation at 4°C overnight, protein G-conjugated beads (25 µl) were added to each tube, followed by subsequent incubation with orbital agitation for 1 h at 4°C. Beads were precipitated as above, and the supernatant (defined at the post-precipitate) was transferred to a new tube. Each pellet (defined as the precipitate) was washed three times by resuspension in 50 mM Tris-HCl (pH 7.5) containing 150 mM NaCl, 5 mM EDTA, 0.1% Triton X-100, and 0.02% SDS with sedimentation between each wash. Samples of each fraction (total lysate, pre-precipitate, precipitate, and post-precipitate) were diluted 1:5 in 187.5 mM Tris-HCl (pH 6.8) containing 6% SDS, 30% glycerol, 0.03% (w/v) bromophenol blue, and 2% BME for SDS-PAGE.

Samples were subjected to SDS-PAGE with 4-15% polyacrylamide Tris-Glycine gels (BioRad, Hercules, CA, USA), and transferred for 1 h to nitrocellulose membranes (Lee and Shea, 2014). The resulting nitrocellulose replicas were blocked in 5% goat serum-1% bovine serum albumin (BSA) in Tris-Buffered Saline containing 0.1% Tween-20 (TBST) with reciprocal shaking for 1 h at room temperature, followed by incubation overnight with one of the following antibodies: a 1:1000 dilution of a mouse monoclonal anti-GFP antibody (Applied Biological Materials, New York City, NY, USA), a 1:500 dilution of a rabbit polyclonal antibody directed against all NF subunits regardless of phosphorylation state (R39; Jung and Shea, 1999), or a 1:100 dilution of a mouse monoclonal antibody directed against a developmentally-delayed NF-H phospho-epitope (RT97, generous gift of Dr B. Anderton, Institute on Psychiatry, King's College, London, UK; Veeranna et al., 2008).

Immunoreactive species were detected with a 1:10,000 dilution of the appropriate alkaline phosphatase-conjugated secondary antibody (Sigma Aldrich) and BCIP/NBT substrate (BioRad). All samples intended for analytical comparison were either run on the same gel, or, in the case of large sample sets, on multiple gels that were electrophoresed, transferred to nitrocellulose, and visualized simultaneously, under identical conditions. Quantification of immunoreactive species was carried out using ImageJ (NIH) software of scanned images of processed nitrocellulose replicas. An average background signal of equivalent area was quantified in two adjacent regions within the same lane and subtracted from quantification of each immunoreactive species (Yabe et al., 2001). GFP-tagged NF-H migrates as multiple phospho-isoforms from 200-260 kDa on SDS gels (e.g. Lee et al., 2014); breakdown products were not included in quantification. R39-immunoreactive species corresponding to the molecular weight of endogenous NF-H (160-200 kDa), but not NF-M (145-160 kDa) or NF-L (70 kDa), were included in analyses.

Statistical analyses were performed using one-way ANOVA with Fisher's post hoc analyses (StatPlus Software). Comparisons with **P<0.05 were considered to be statistically significant; comparison with *P<0.1 but >0.05 were considered to represent a trend towards significance. For presentation, lines within a single treatment condition indicate which columns are being compared; in instances where no connecting lines are present, asterisk(s) indicate comparison of that condition versus the corresponding untreated control condition.

### Immunocytochemistry

Cells were cultured on laminin and poly-D-lysine-coated coverslips and co-transfected with shRNA directed against NF-H and constructs expressing wt-H, S493A, or S493D as described above (Lee et al., 2014). Cultures were washed briefly with phosphate buffered saline (PBS, pH 7.4) and fixed for 15 min at room temperature with 4% paraformaldehyde in PBS. Cultures were then washed three times with PBS (5 min/wash) and blocked in 10% goat serum in PBS containing 0.2% Triton X-100 for 1 h at room temperature. Cultures were incubated overnight at 4°C with a 1:100 dilution of RT97 in 2% goat serum in PBS with 0.2% Triton X-100. Cultures were washed three times with PBS (5 min/wash) and then incubated for 1 h at room temperature in rhodamine red-conjugated goat anti-mouse antibody (1:500) in 2% goat serum-PBS with 0.2% Triton X-100. Cultures were washed three times with PBS (5 min/wash). Water was removed from the cultures by sequential rinses in a series of increasing ethanol concentrations (70, 80, 95, 100%). Coverslips were then rinsed twice in xylene and mounted on glass slides using the non-aqueous mounting medium DePeX, and allowed to dry overnight.

Fluorescent intensity was quantified within axonal neurites and soma. RT97 immunoreactivity within perikarya versus axonal neurites were compared as an index of NF phosphorylation level and relative distribution of phospho-H. The percentage of cells containing perikaryal GFP and phospho-H aggregates was also quantified. Cells containing non-diffuse tanged/aggregated immunoreactivity within ≥50% of their perikarya were scored as ‘possessing aggregates’ or ‘aggregated’. Kinesin immunoreactivity was compared as an index of distribution of this motor protein within perikarya and neurites by calculating a ratio of somal/neurite densitometric levels in cells displaying GFP (as confirmation of expression of respective constructs). In efforts to approximate the consequence of expression of NF construct on differential distribution of kinesin immunoreactivity, we also quantified this ratio for cells within cultures transfected with S493D but expressing no detectable GFP signal. These cells were considered to represent the distribution of kinesin in the absence of detectable expression of NF constructs, and in additional analyses this ratio was subtracted from the ratio of all values of transfected cells. Statistical analyses were performed using one-way ANOVA with Fisher's post hoc analysis (StatPlus Software).
